# Exome Sequencing Identifies Three Novel Candidate Genes Implicated in Intellectual Disability

**DOI:** 10.1371/journal.pone.0112687

**Published:** 2014-11-18

**Authors:** Zehra Agha, Zafar Iqbal, Maleeha Azam, Humaira Ayub, Lisenka E. L. M. Vissers, Christian Gilissen, Syeda Hafiza Benish Ali, Moeen Riaz, Joris A. Veltman, Rolph Pfundt, Hans van Bokhoven, Raheel Qamar

**Affiliations:** 1 Department of Biosciences, Faculty of Science, COMSATS Institute of Information Technology, Islamabad, Pakistan; 2 Department of Human Genetics, Nijmegen Centre for Molecular Life Sciences, Radboud University Medical Centre, Nijmegen, the Netherlands; 3 Department of Bioinformatics and Biotechnology, International Islamic University, Islamabad, Pakistan; 4 Department of Cognitive Neurosciences, Donders Institute for Brain, Cognition and Behaviour, Nijmegen, The Netherlands; 5 Department of Biochemistry, Al-Nafees Medical College & Hospital, Isra University, Islamabad, Pakistan; University of Heidelberg, Germany

## Abstract

Intellectual disability (ID) is a major health problem mostly with an unknown etiology. Recently exome sequencing of individuals with ID identified novel genes implicated in the disease. Therefore the purpose of the present study was to identify the genetic cause of ID in one syndromic and two non-syndromic Pakistani families. Whole exome of three ID probands was sequenced. Missense variations in two plausible novel genes implicated in autosomal recessive ID were identified: lysine (K)-specific methyltransferase 2B (*KMT2B*), zinc finger protein 589 (*ZNF589*), as well as hedgehog acyltransferase (*HHAT*) with a *de novo* mutation with autosomal dominant mode of inheritance. The *KMT2B* recessive variant is the first report of recessive Kleefstra syndrome-like phenotype. Identification of plausible causative mutations for two recessive and a dominant type of ID, in genes not previously implicated in disease, underscores the large genetic heterogeneity of ID. These results also support the viewpoint that large number of ID genes converge on limited number of common networks i.e. ZNF589 belongs to KRAB-domain zinc-finger proteins previously implicated in ID, HHAT is predicted to affect sonic hedgehog, which is involved in several disorders with ID, *KMT2B* associated with syndromic ID fits the epigenetic module underlying the Kleefstra syndromic spectrum. The association of these novel genes in three different Pakistani ID families highlights the importance of screening these genes in more families with similar phenotypes from different populations to confirm the involvement of these genes in pathogenesis of ID.

## Introduction

Intellectual disability (ID), a neurocognitive disorder, is characterized by substantial limitations both in intellectual functioning and in adaptive behavior. In patients diagnosed before the age of 18 years, it has a prevalence of 2–3% in the general population [1]. Stein et al. [2] reported the prevalence of ID among 3–9 years old children in different populous countries including Pakistan, Brazil, India, Bangladesh and Philippines, which varied from 9/1000 to 156/1000. ID is an unsolved healthcare problem, which creates an enormous socioeconomic burden on the society, especially in the underdeveloped countries where there is a high rate of consanguinity, resulting in further aggravating the genetically inherited disease prevalence [3]. Chromosomal abnormalities and single gene disruptions contribute significantly to all forms of ID, including severe, moderate and mild phenotype [4]. Investigations aiming to unravel the genetic defects initially focused mainly on X-linked ID since male ID patients are overrepresented as compared to females with a ratio of 1∶1.3 to 1∶1.9 [5]. However, these investigations have revealed that only 10% of ID cases are due to X chromosomal defects while the remaining cases are expected to be caused by genetic defects in the autosomes and equally due to adverse environmental effects such as poor mother health, social deprivation, infections and injuries during prenatal life and hypoxia [6]. Chromosomal aberrations and mutations in more than 450 genes can explain the disorder in about half of all ID patients [5]. The large number of ID genes present a challenge for the identification of the genetic defect in individual families and isolated cases, however, only a limited number of pathways are emerging whose disruption appears to be shared by groups of ID genes [7].

Despite being clinically heterogeneous, syndromic ID (sID) as well as non-syndromic ID (nsID) share common neurological features, such as autism, epilepsy, ADHD (attention deficit hyperactivity disorder) and behavioral anomalies [7]. In addition, syndromic forms of ID are characterized by a pattern of congenital anomalies that can be seen in addition to ID and other neurological features. The latter can help to establish clinical diagnosis, which can subsequently be validated by direct molecular diagnostic testing of targeted gene(s). However, the number of syndromes where similar phenotypes can be caused by mutations in a variety of different genes is increasing. Examples of these include Bardet-Biedl syndrome (MIM 209900; 17 genes), Sotos syndrome (MIM 117550; 2 genes) and Kleefstra syndrome (MIM 610253; 5 genes) [7]–[10]. Typically, the underlying genes for each of these syndromes have a functional relationship to each other and mutations lead to disruption of the same molecular pathways. As in the case of Kleefstra syndrome (KS), which is characterized by severe to moderate ID, speech impairment, congenital hypotonia, specific distinguishing facial features and complex pattern of other anomalies can be caused by *de novo* mutations affecting epigenetic regulators such as euchromatic histone-lysine N-methyltransferase 1 (*EHMT1*), lysine (K)-specific methyltransferase 2C (*KMT2C*), SWI/SNF related, matrix associated, actin dependent regulator of chromatin, subfamily b, member 1 (*SMARCB1*), nuclear receptor subfamily 1, group I, member 3 (*NR1I3*) and methyl-CpG binding domain protein 5 (*MBD5*) [11]–[13].

Till now, small families with ID have not been studied extensively due to technical difficulties including non-suitability of homozygosity mapping and use of simple traditional linkage analysis with insufficient power to resolve the genetic cause in such families. The genetic analyses combined with whole exome sequencing have recently enabled the systematic identification of pathogenic mutations in small families with recessive nsID and sID, including mutations in 13 novel nsID genes [13]. Najamabadi et al. [4] have recently proposed 29 candidate genes for autosomal recessive nsID. In the current study missense homozygous mutations in two novel genes including lysine (K)-specific methyltransferase 2B (*KMT2B*), a zinc finger gene *ZNF589* and a heterozygous *de novo* mutation in hedgehog acetyltransferase (*HHAT*) were identified.

## Methods

### Ethics statement

This study was approved by the Department of Biosciences Ethics Review Board of the COMSATS Institute of Information Technology, Islamabad, Pakistan, and the local Ethics Committee of the Radboud University Medical Centre, Nijmegen, The Netherlands. All family members and 200 ethnically matched control individuals were informed about the purpose of the study and written consent in their local language was taken before recruitment and sampling. The parents or guardians of the individuals in this manuscript have given written consent (as outlined in PLOS consent form) to publish the patients case details.

### Clinical features

**Figure 1:**
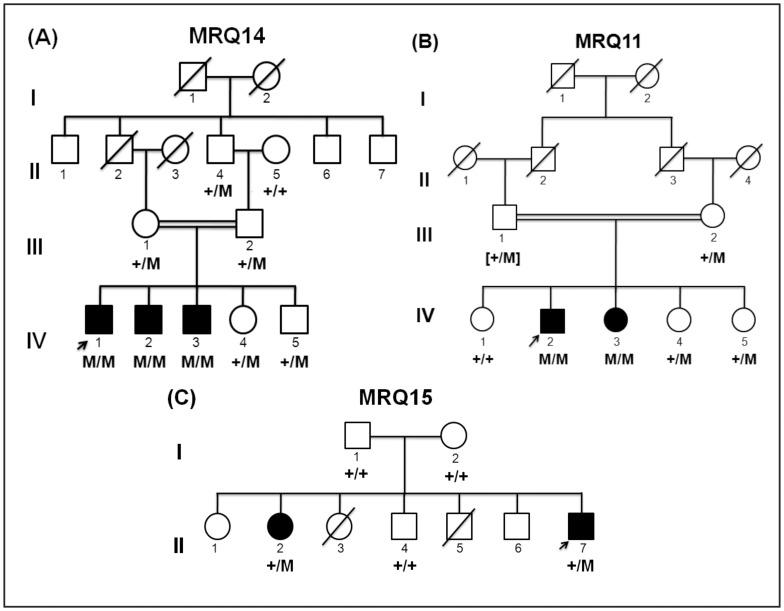
Pedigree of families (A) MRQ14, (B) MRQ11 and (C) MRQ15. The segregation of mutation of *KMT2B*, *ZNF589* and *HHAT* are also in the pedigree. The symbol +/+ represents homozygous ancestral alleles, M/M is for homozygous variant alleles and +/M is for heterozygous carriers. In the panel B, the genotype of the father (III:1) has been deduced.

**Figure 2:**
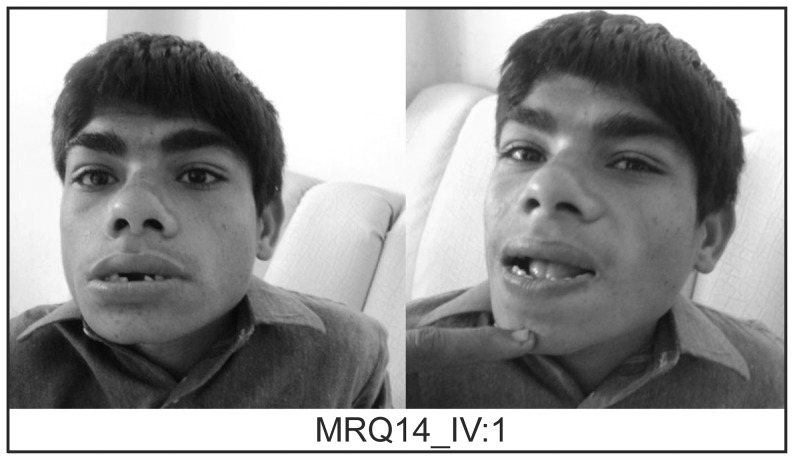
Photographs of MRQ14 proband. The photographs demonstrate the classic facial features representative of Kleefstra syndrome.

A consanguineous family MRQ14 (Figure 1A) with three children with severe sID was sampled from central Punjab, Pakistan, including three affected sons (IV:1, IV:2, IV:3) unaffected daughter and son (IV:4 and IV:5), the unaffected mother (III:1), unaffected father (III:2), unaffected paternal grandfather (II:4) and unaffected paternal grandmother (II:5). The three affected brothers had a similar sID phenotype and were each born after about 39 weeks of uneventful pregnancies with normal labor. At the age of 16 years, the proband (IV:1) had short stature, dysmorphic facial features including a large head, flattened nasal bridge, apparent hypertelorism synophrys, midface hypoplasia, thick eyebrows, everted lower lip, dental anomalies and prognathism. Upon physical examination, he was found to suffer from musculoskeletal anomalies as well as cryptorchidism and micropenis. Being floppy during childhood he was found to be hypotonic on reaching teenage (Figure 2). In addition to speech and motor delay, he was underweight at the time of assessment (23kg, 2^nd^ centile) and was short for his age (81cm, 2^nd^ centile). All the three affected boys lacked language development, had profound ID (IQ<20, IQ was tested using the Wechsler Intelligence Scale for Children (WISC-III)), and they were not able to perform essential activities of daily life. Computed Tomography (CT) scan did not reveal any anomaly of the brain and the biochemical tests including liver transaminases, serum lactate as well as serum electrolyte and complete blood count were all in the normal ranges (Table 1).

**Table 1:** Clinical features of Kleefstra syndrome shared by the three affected brothers of family MRQ14.

Clinical features	Patient-IV:1 (Proband)	Patient-IV:2	Patient-IV:3
High birth weight	no	no	no
Microcephaly	no	no	no
Synophrys	yes	yes	yes
Unusual shape of the eyebrows	yes	yes	yes
Midface hypoplasia	yes	yes	yes
Full everted lower lip	yes	yes	yes
Cupid bowed upper lip	yes	yes	yes
Protruding tongue	yes	yes	yes
Prognathism	yes	yes	yes
Short stature	yes	yes	yes
Overweight (BMI>25)	no	no	no
DD/ID	yes (severe)	yes (severe)	yes (severe)
Heart defect	no	no	no
Genital anomaly	yes	yes	yes
Renal anomaly (including VUR)	no	no	no
Recurrent infections	yes	yes	yes
Hearing deficit	no	no	no
Gastro-esophageal reflux	no	no	no
Epilepsy	yes	yes	yes
Behavioral/psychiatric problems	yes	yes	yes
Anomalies on brain imaging	no	not performed	not performed
Tracheomalacia	no	no	no
Umbilical/inguinal hernia	no	no	no
Anal atresia	no	no	no
Musculoskeletal anomaly	yes	yes	yes
Respiratory complications	no	no	no
Hypertelorism	yes	yes	yes

BMI, body mass index; DD, developmental disability; ID, intellectual disability, VUR, vesico-ureteric reflux

The consanguineous family MRQ11 with two affected children (Figure 1B) was sampled from Northern Pakistan. The sampled members were: unaffected mother (III:2), unaffected daughters (IV:1, IV:4, IV:5), affected son (IV:2), and affected daughter (IV:3). The two affected siblings had a similar nsID phenotype. The affected members, a son (IV:2), and a daughter (IV:3) were born after an uneventful 39 weeks pregnancy. Labor was normal and they did not undergo postnatal hypoxia. Both of them had delayed milestones including speech and motor development. At the time of examination, at the ages of 14 years (IV:2) and 10 years (IV:3), they were both below average weight and height: the affected son was 23 kg and 114 cm (both 2^nd^ centile), whereas the affected daughter weighed 22 kg and was 88 cm in height (both being 2^nd^ centile). Both siblings had strabismus and had moderate ID (IQ: 36–51). CT scan did not show any anomaly of the brain, in addition, metabolic and biochemical testing revealed no abnormalities.

Family MRQ15 was a non-consanguineous family (Figure 1C), from Punjab, Pakistan. Five members of the family were sampled that included an unaffected father (I:1), unaffected mother (I:2), affected daughter (II:2), unaffected son (II:4) and affected son (II:7). The two affected siblings had profound ID (IQ<20) and could speak only a few meaningful words and could recognize only their parents and siblings. They were both not able to perform daily activities of life independently. No other syndromic features were present and their CT scan did not show any brain malformation.

### Homozygosity mapping and CNV analysis

The pedigrees are concordant with the recessive inheritance in all the three families, therefore in order to obtain copy number variation (CNV) as well as homozygosity mapping data, Affymetrix 250K NSPI SNP (Affymetrix, Santa Clara, CA) array analysis was performed for MRQ14 family members IV:1, IV:2, IV:3 and IV:5, and all sampled affected and unaffected members of MRQ11 and MRQ15 (Figure 1). For CNV determination, the data were scrutinized using Copy Number Analyzer for GeneChip [14]. Affymetrix Genotyping Console (version 2.0) was used to obtain the genotype data and online software Homozygosity Mapper [15] was used to obtain the homozygous regions. The homozygous intervals of at least 1 Mb were also verified visually. All data were mapped using the Human Genome Build hg19.

### Exome sequencing

Homozygosity mapping and CNV analysis did not reveal the genetic cause of the disease in any of the family therefore exome sequencing was performed for the proband of each family. The exomes of the probands were enriched and sequenced as described previously by Vissers et al. [16]. In brief, using an Agilent SureSelect Human All Exon Kit (50 Mb, ∼21,000 genes; Agilent Technologies, Santa Clara, CA) exome libraries were prepared as described by the manufacturer and pooled for bead amplification using the emulsion-based clonal PCR (emPCR) of EZbead system (Life Technologies, Santa Clara, CA) and were subsequently sequenced using the SOLiD 4 system (Life Technologies, Santa Clara, CA). As described by Vissers et al. [16] the variants and indels were only selected for further analysis when the overall variant reads were at least 15% of the total number of reads, with a minimum of 5 reads [17]. For each proband, 36,001 to 44,200 variants were annotated. All nongenic, non-splice site, intronic and synonymous variants were excluded from further analysis. Furthermore, the low frequency variants (less than 0.5%) in known ID genes present in dbSNP were also checked, such variants were further filtered on the basis of pathogenicity scores and tested for segregation among the respective family members while all the other variants were excluded (data not shown), which were found in dbSNP132 or those in an in-house database. To predict the pathogenicity of the variants, data were evaluated using *in silico* analysis, including Poly Phen-2 (genetics.bwh.harvard.edu/pph2) and SIFT (**s**ift.jcvi.org/).

### Sanger sequencing

Sanger sequencing for confirmation of the candidate gene variants and their segregation in the families was performed (Tables 2, 3 and 4) by designing PCR primers (Table S1, S2 and S3) using the Primer3 program (http://frodo.wi.mit.edu/) and amplifying the regions of interest. PCR amplification was conducted using 0.25 mM dNTPs, 1X PCR buffer (100 mM Tris-HCl, pH 8.3, 500 mM KCl), 2.5 mM Mg^+2^, 0.5 µM of each primer, 2.5 U *Taq* polymerase (Fermentas Life Sciences, Ontario, Canada) and 50 ng gDNA. The thermal profile consisted of initial denaturation at 95°C for 5 min followed by 30 cycles of amplification at 95°C for 1 min, 57°C for 30 sec and 72°C for 45 sec, a final extension was carried out at 72°C for 5 min. Purified PCR amplicons were then sequenced using the ABI PRISM Big Dye Terminator Cycle Sequencing V3.1 ready reaction kit and the ABI PRISM 3730 DNA analyzer (Applera Corp, Foster City, CA).

**Table 2:** Family MRQ14 homozygous and compound heterozygous variant validation using Sanger sequencing and *in silico* pathogenecity predictions.

Gene (NM ID)	Protein function	cDNA change	Amino acid change	PhyloP score	Grantham distance	SIFT	Mutation taster	Polyphen2	Zygosity	Segregation in family
***NME7*** ** (NM_013330.4)**	Unknown	c.38C>T	p.(Arg13Gln)	5.53	43	Deleterious	Disease causing	Probably damaging	Homozygous	No
***PPP1R9A*** ** (NM_001166160.1)**	Nervous system development	c.1387C>T	p.(Pro463Ser)	5.29	74	Deleterious	Disease causing	Probably damaging	Homozygous	No
***DYM*** ** (NM_017653.3)**	Dyggve-melchior-clausen disease, 223800 (3); smith-mc-Cort dysplasia,	c.1205A>T	p.(Leu402*)	4.571	1000	Unknown	Unknown	Unknown	Homozygous	No
***DLG1*** ** (NM_004087.2)**	Cell to cell adhesion, nervous system involvement	c.574T>C	p.(Ile192Val)	4.518	29	Deleterious	Disease causing	Probably damaging	Homozygous	No
***KMT2B*** ** (** ***MLL4*** **) (NM_014727.1)**	Unknown	c.2456C>T	p.(Pro819Leu)	4.429	98	Tolerated	Disease causing/polymorphism	Probably damaging	Homozygous	Yes
***EHMT2*** ** (NM_006709.3)**	Chromatin modification, biological process and nervous system involvement	c.1151C>T	p.(Arg384Gln)	3.503	43	Deleterious	Disease causing	Probably damaging	Homozygous	No
***VAV2*** ** (NM_001134398.1)**	Nervous system phenotype	c.2495A>G	p.(Met832Thr)	3.138	81	Deleterious	Disease causing	Probably damaging	Homozygous	No
***PLCD4*** ** (NM_032726.2)**	Intercellular signaling cascade	c.1885C>T	p.(Leu629Phe)	2.9	22	Deleterious	Disease causing	May be damaging	Homozygous	No
***ZNF227*** ** (NM_182490.2)**	Regulation of transcription, DNA-dependent	c.956C>T	p.(Thr319Ile)	2.715	89	Deleterious	Polymorphism	May be damaging	Homozygous	No
***SMEK1*** ** (NM_032560.4)**	Unknown	c.2239A>C	p.(Ser747Ala)	3.03	98	Tolerated	Disease causing	Not damaging	Homozygous	No
***UGT8*** ** (NM_001128174.1)**	CNS development	c.359A>G	p.(Asn120Ser)	2.47	46	Tolerated	Disease causing	Not damaging	Homozygous	No
***DNAH17*** ** (NM_173628.3)**	Microtubule-based movement, ciliary or flagellar motility	c.12599A>C	p.(Val4200Gly)	4.821	109	Deleterious	Unknown	Probably damaging	Heterozygous	No
***DNAH17*** ** (NM_173628.3)**	Microtubule-based movement, ciliary or flagellar motility	c.12267C>T	p.(Met4089Ile)	5.974	10	Deleterious	Unknown	Probably damaging	Heterozygous	No
***SACS*** ** (NM_014363.5)**	Protein folding	c.10291C>G	p.(Val3431Leu)	4.266	32	Deleterious	Disease causing	Probably damaging	Heterozygous	No
***SACS*** ** (NM_014636.2)**	Protein folding	c.5461A>G	p.(Cys1821Arg)	2.631	180	Deleterious	Disease causing	Probably damaging	Heterozygous	No
***TEP1*** ** (NM_007110.4)**	Telomere maintenance via recombination	c.3519>C	p.(Lys1174Glnfs*16)	2.109	1000	Unknown	Unknown	Unknown	Heterozygous	No
***TEP1*** ** (NM_007110.4)**	Telomere maintenance via recombination	c.1817G>A	p.(Pro606Leu)	3.26	98	Tolerated	Polymorphism	Not damaging	Heterozygous	No

NM, mRNA accession number; PhyloP, Phylogenetic P-values; Polyphen, Polymorphism phenotyping; SIFT, Sorting intolerance from tolerance, Mutation taster (http://www.mutationtaster.org).

**Table 3:** Family MRQ11 homozygous and compound heterozygous variant validation using Sanger sequencing and *in silico* prediction.

Gene (NM ID)	Protein function	cDNA change	Amino acid change	PhyloP	Grantham distance	SIFT	Mutation taster	Polyphen2	Zygosity	Segregation in family
***SMOX*** ** (NM_175839.2)**	Oxidation reduction	c.1604C>A	p.(Ser535Tyr)	5.409	144	Deleterious	Disease causing	Probably damaging	Homozygous	No
***TAS1R2*** ** (NM_152232.2)**	Signal transduction	c.971C>T	p.(Gly324Asp)	5.23	94	Deleterious	Disease causing	Probably damaging	Homozygous	No
***ATP11A*** ** (NM_015205.2)**	ATP biosynthetic process	c.64G>T	p.(Asp22Tyr)	5.14	160	Deleterious	Disease causing	Probably damaging	Homozygous	No
***ADORA2B*** ** (NM_000676.2)**	Positive regulation of cAMP biosynthetic process	c.590T>C	p.(Ile197Thr)	4.87	89	Deleterious	Disease causing	Probably damaging	Homozygous	No
***ZNF589*** ** (NM_016089.2)**	Regulation of transcription, DNA-dependent	c.956T>A	p.(Leu319His)	2.965	100	Deleterious	Polymorphism	Probably damaging	Homozygous	Yes
***ZNF502*** ** (NM_033210.4)**	Unknown	c.746G>A	p.(Arg249His)	1.425	29	Tolerated	Not disease causing	Probably not damaging	Homozygous	No
***ADHFE1*** ** (NM_144650.2)**	Oxidation reduction	c.955A>G	p.(Ile319Val)	3.51	29	Tolerated	Disease causing	Probably not damaging	Heterozygous	No
***ADHFE1*** ** (NM_144650.2)**	Oxidation reduction	c.1172C>T	p.(Thr391Ile)	1.26	89	Tolerated	Disease causing	Probably damaging	Heterozygous	No
***CMYA5*** ** (NM_153610.3)**	Unknown	c.7472T>C	p.(Ile2491Thr)	2	89	Deleterious	Disease causing	Probably damaging	Heterozygous	No
***CMYA5*** ** (NM_153610.3)**	Unknown	c.8534G>A	p.(Arg2845Lys)	0.51	26	Tolerated	Not disease causing	Probably not damaging	Heterozygous	No
***DCHS1*** ** (NM_003737.2)**	Calcium-dependent cell-cell adhesion	c.3017C>T	p.(Arg1006His)	2.55	29	Deleterious	Disease causing	Probably damaging	Heterozygous	No
***DCHS1*** ** (NM_003737.2)**	Calcium-dependent cell-cell adhesion	c.1265T>A	p.(Tyr422Phe)	4.91	22	Deleterious	Disease causing	Probably damaging	Heterozygous	No
***DPAGT1*** ** (NM_001382.3)**	UDP-N-acetylglucosamine metabolic process	c.951G>C	p.(Ser317Arg)	4.39	110	Deleterious	Disease causing	Probably damaging	Heterozygous	No
***DPAGT1*** ** (NM_001382.3)**	UDP-N-acetylglucosamine metabolic process	c.38A>T	p.(Ile13Asn)	4.86	149	Deleterious	Disease causing	Probably damaging	Heterozygous	No
***DENND2C*** ** (NM_001256404.1)**	Unknown	c.1129T>C	p.(Lys377Glu)	3.608	56	Unknown	Unknown	Unknown	Heterozygous	No

NM, mRNA accession number; PhyloP, Phylogenetic P-values; Polyphen, Polymorphism phenotyping; SIFT, Sorting intolerance from tolerance, Mutation transfer (http://www.mutationtaster.org)

**Table 4:** Family MRQ15 homozygous and compound heterozygous variants validation using Sanger sequencing and *in silico* prediction.

Gene (NM ID)	Protein function	cDNA change	Amino acid change	PhyloP	Grantham distance	SIFT	Mutation taster	Polyphen2	Zygosity	Segregation in family
***SF3B3*** ** (NM_012426.2)**	RNA splicing	c.82C>A	p.(Gln28Lys)	6.119	53	Deleterious	Disease causing	Probably damaging	Homozygous	No
***LARGE*** ** (NM_004737.4)**	N-acetylglucosamine metabolic process	c.251C>G	p.(Ser84Thr)	5.72	58	Deleterious	Disease causing	Probably damaging	Homozygous	No
***HHAT*** ** (NM_001122834.2)**	Multicellular organism development	c.1158G>C	p.(Trp386Cys)	5.433	215	Deleterious	Disease causing	Probably damaging	Heterozygous	Present in affected only
***NEDD4*** ** (NM_198400.1)**	Protein ubiquitination during ubiquitin-dependent protein catabolic process	c.872C>T	p.(Gly291Glu)	2.915	98	Deleterious	Polymorphism	Probably damaging	Homozygous	No
***PLCH1*** ** (NM_001130960.1)**	Cell division	c.13+1C>T	No	4.407	0	Unknown	Unknown	Unknown	Homozygous	No
***PDS5B*** ** (NM_015032.3)**	Intercellular signaling cascade	c.25-1G>A	No	6.172	0	Unknown	Unknown	Unknown	Homozygous	No
***WASF1*** ** (NM_003931.2)**	Protein complex assembly	c.4+1C>T	No	5.63	0	Unknown	Unknown	Unknown	Homozygous	No
***DENND2A*** ** (NM_015689.3)**	Unknown	c.54G>C	p.(Pro297Arg)	2.342	110	Deleterious	Disease causing	Probably damaging	Homozygous	No
***HMCN1*** ** (NM_031935.2)**	Response to stimulus	c.7163G>A	p.(Gly2388Glu)	4.013	98	Deleterious	Disease causing	Probably damaging	Heterozygous	No
***HMCN1*** ** (NM_031935.2)**	Response to stimulus	c.13190G>A	p.(Arg4397Gln)	0.754	43	Tolerated	Disease causing	Probably not damaging	Heterozygous	No
***MED13L*** ** (NM_015335.4)**	Regulation of transcription from RNA polymerase II promoter	c.1447G>T	p.(Pro483Thr)	3.577	38	Tolerated	Disease causing	Probably not damaging	Heterozygous	No
***MED13L*** ** (NM_015335.4)**	Regulation of transcription from RNA polymerase II promoter	c.740A>G	p.(Leu247Pro)	2.644	98	Tolerated	Disease causing	Probably not damaging	Heterozygous	No
***ZNF772*** ** (NM_001024596.2)**	Regulation of transcription, DNA-dependent	c.1145T>G	p.(Glu382Ala)	2.468	107	Tolerated	Disease causing	Probably not damaging	Heterozygous	No
***ZNF772*** ** (NM_015335.4)**	Regulation of transcription, DNA-dependent	c.878G>T	p.(Pro293His)	1.828	77	Deleterious	Polymorphism	Probably damaging	Heterozygous	No

NM, mRNA accession number; PhyloP, Phylogenetic P-values; Polyphen, Polymorphism phenotyping; SIFT, Sorting intolerance from tolerance, Mutation taster (http://www.mutationtaster.org).

## Results

**Figure 3:**
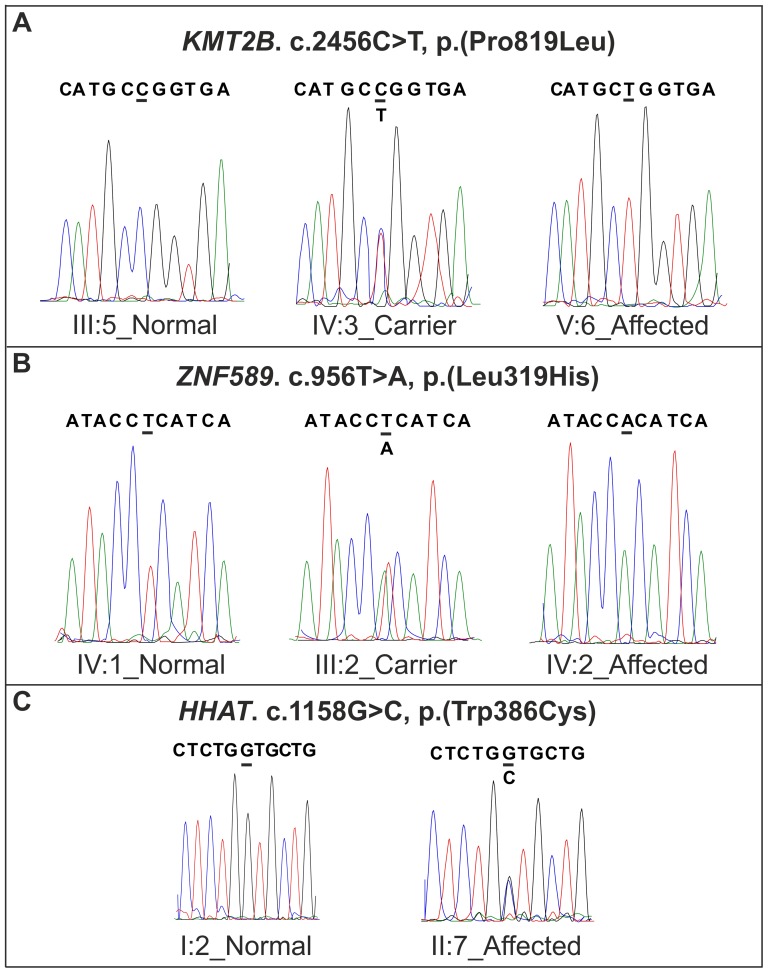
The sequencing chromatograms of the families MRQ14, MRQ11 and MRQ15. (**A**) Shows the panels containing the region with the identified *KMT2B* mutation in family MRQ14: ancestral (left panel), heterozygous (middle panel) and variant (right panel) (**B**) shows the region containing the identified *ZNF589* mutation in family MRQ11: ancestral (left panel), heterozygous (middle panel) and variant (right panel). (**C**) shows the *de novo* variant of *HHAT* in family MRQ15: ancestral (left panel), heterozygous (right panel).

After filtering the exome data of the proband (IV:1) of family MRQ14 (Figure 1A), assuming a recessive inheritance model, 53 homozygous and 11 compound heterozygous variants in different genes were obtained. The data were further prioritized to get the most relevant changes using a phyloP score>2.0. This resulted in 11 homozygous and 6 compound heterozygous variants in 14 different genes (Table 2). A variant in *KMT2B* (chr19.hg19:g.36,208,921_36,229,779), c.2456C>T, p.(Pro819Leu), was the only variant in the shared homozygous region that segregated with the disease in the family after Sanger sequencing (Figure 1A and 3A). In addition, the variant was absent in 200 age and ethnicity-matched control samples (n = 400 alleles) as well as in families MRQ11 and MRQ15. The *KMT2B* c.2456C>T mutation segregated with the disease in the family with the change inherited by descent from the heterozygous paternal grandfather (III:4), the maternal grandparents were not available for screening of the change, but it is likely that the other mutant allele was inherited from the maternal grandfather as the parents are first cousins.

Exome sequencing of the proband of family MRQ11 resulted in the identification of 38 homozygous and 30 compound heterozygous changes in 38 and 15 genes. Homozygosity mapping revealed only four common homozygous regions between the 2 affected members of the family (Table S4). The data were further prioritized (as described above), which revealed a total of 15 variants in 11 different genes (7 homozygous and 8 compound heterozygous; Table 3). These variants were further validated by Sanger sequencing for segregation in the respective family. Variant c.956T>A, p.(Leu319His) in *ZNF589* (chr3.hg19:g.48,282,596_48,329,115) segregated with the phenotype in the family (Figure 1B and 3B) and was absent in the control population as well as families MRQ14 and MRQ15.

Genotyping of family MRQ15 by microarray analysis did not reveal any homozygous region shared by the affected members, suggesting that compound heterozygosity as well as *de novo* dominant mutation may cause the disease in this family. Whole exome sequencing was carried out to identify the genetic cause of ID in this family using a trio-based approach [18], [19]. After variant filtration as described above, 8 homozygous and 6 compound heterozygous variants were identified in 11 different genes, which were further screened in the family by Sanger sequencing (Table 4). However, none of these selected variants segregated with the disease. In-line with a hypothesized dominant *de novo* mutation model, a heterozygous change c.1158G>C, p.(Trp386Cys) in the *HHAT* was present in both the affected members (Figure 1C and 3C) and absent in the parents and the unaffected sibling as well as healthy controls, and families MRQ14 and MRQ11. The occurrence of the mutation in both affected members is consistent with a germline mosaicism in one of the unaffected parents. Of note, non-paternity was excluded by segregation analysis of rare paternal variants. It was not possible to obtain other tissues of the parents therefore presence of variants could not be checked in those tissues.

## Discussion

Genetic screening of three Pakistani ID families in the current study resulted in the identification of three novel plausible ID genes, *KMT2B*, *ZNF589* and *HHAT*. The phenotype of the three affected members of family MRQ14 had Kleefstra syndrome-like phenotype (clinical details are described in clinical features section), which is characterized by facial dysmorphism, hypotonia and mild to severe ID (Figure 2). This syndrome is rare with unknown prevalence and has not been reported in Pakistan previously. The homozygous variant c.2456C>T; p.(Pro819Leu) identified in the current study in the *KMT2B* was found to segregate in the family in a recessive pattern.

*KMT2B* belongs to the MLL (myeloid/lymphoid or mixed-lineage leukemia) family, and was found to be ubiquitously expressed in adult tissues as well as in solid tumor cell lines [19], its involvement in human cancer has already been established. Furthermore, *KMT2B* has been reported to express in different parts of human brain such as medial frontal cortex, occipital cortex, hippocampal cortex, amyloid complex and basal ganglia at different ages ranging from 0 to 48 months (Allen Institute for Brain Science. Allen Human Brain Atlas (http://human.brain-map.org/)). The protein encoded by *KMT2B/MLL4* has multiple domains such as a CXXC zinc finger, three PHD zinc fingers, a SET (suppressor of variegation, enhancer of zeste, and trithorax) and two FY domains. Of all the domains, the SET domain is highly conserved in KMT2B and is a hallmark of the KMT gene family. Kleefstra et al. [12] reported that the disease in 25% of the patients with KS was caused by haploinsufficiency of *EHMT1*, while the disease in few other patients diagnosed with a similar phenotype was explained by *de novo* mutations in *MBD5*, *SMARCB1*, *NR1I3* and *KMT2C* [8]. Kleefstra et al. [8], reported a *de novo* mutation in *KMT2C* to cause dominant ID, similarly 41 likely pathogenic mutations in *KMT2D* gene causing Kabuki syndrome (MIM 147920) have been reported in 86 patients, having dysmorphic facial features, bone deformities, hypotonia, congenital heart defects, ID, urinary tract and respiratory tract infections [20]. Kerimogulo et al. [21] have recently reported that *KMT2B*/*MLL2* belongs to myeloid leukemia gene family, which mediates hippocampal histone 3 lysine 4 di- and trimethylation in memory formation, thus their data supports the *KMT2B*/*MLL4* involvement along with *KMT2B*/*MLL2* in cognition [22]. These findings support the current results that mutations in *KMT2B*/*MLL4* could possibly lead to a Kleefstra syndrome-like phenotype. The current work is the first report of a recessive KS finding, involving the KMT gene family, which has already been implicated in dominant forms of KS and Kabuki syndrome. The identified variant was excluded in 200 age and ethnicity matched controls. KMT genes (*KMT2A*-*KMT2E*) as well as their *Drosophila* orthologs; trithorax (trx) and trithorax related (trr), express protein products, which are capable of methylating histone H3 on lysine 4 (H3K4). Of note, the *Drosophila trr* gene is the single ortholog of mammalian *KMT2C* and *KMT2B*, and has been shown to be involved in cell proliferation but its mechanism of action is not yet known [23]. However, its ablation results in restricted tissue growth, which explains the growth retardation in mouse model with low levels of *KMT2B* and could also, be the reason for growth delays in affected members in the current study. KMT2B, like KMT2C is a part of the ASCOM (activating signal cointegrator-2) co-activator complex, which has an important role in epigenetic regulation together with the nuclear-receptor transactivation and forms a connection between the two complexes [12]. Kim et al. [23] have previously reported that ASCOM-KMT2B plays an essential role in Farnesoid X receptor trans-activation through their H3K4 trimethylation activity [23]. Hence, it can be proposed that KMT2B could also be a part of the chromatin modification module proposed by Kleefstra et al. [12], along with *KMT2C, SMARCB1* and *NR1I3, MBD5* and *EHMT1*. It is remarkable that the Kleefstra syndrome-like phenotype could be the result of recessive variant identified in family MRQ14 (Figure 1A), whereas all other previously reported gene mutations associated with KS are dominantly inherited. This may reflect an intrinsic property of *KMT2B*, making it less dosage-sensitive than the other KS genes. However, the subsequent functional analyses are highly warranted in order to establish the pathogenicity of the identified recessive mutation.

The phenotypic features of MRQ11 were nsID, however, no striking dysmorphic facial features or structural brain anomalies were observed. The IQ was in the range of moderate ID (IQ: 36-51). Exome sequencing identified a substitution at c.956T>A (p.(Leu319His)) in *ZNF589*, which segregated with the disease in the family in a recessive manner. The gene has not been reported previously for ID and is predicted to be involved in DNA dependent regulation of transcription (Gene ontology database: www.geneontology.org). Based on the brain atlas, *ZNF589* has been shown to be expressed at different ages ranging from 0 to 48 months in different parts of brain such as amyloid complex, medial frontal cortex, hippocampal cortex and basal ganglia. (Allen Institute for Brain Science. Allen Human Brain Atlas; http://human.brain-map.org/). The variant is located in the zinc finger C2H2 domain and *in silico* analysis predicted that the two amino acids differ from each other with a Grantham distance of 100, which depicts a moderate physiochemical difference. The SIFT and polyphen2 also describe this variant to be pathogenic in nature (Table 4). *ZNF589* is localized at the cytogenetic band 3p21.31, it belongs to the kruppel C2H2-type zinc-finger protein family. *ZNF589* consists of a conserved KRAB domain at the amino terminus and four zinc fingers of the C2H2 type at the carboxy terminus. Upon alternative splicing of *ZNF589*, two products are obtained that encode a protein of 361 and 421 amino acids, which differ from each other at the carboxy terminus [24]. The missense change c.956T>A is located in the domain C2H2-type. To date, limited functional data of *ZNF589* is available. Liu et al. [24] have shown that *ZNF589* is involved in hematopoiesis, because of its localization in the bone marrow derived stem cells. The zinc finger genes are housekeeping genes such as the *ZNF589*, which is expressed in the bone marrow stem cell and is involved in DNA dependent transcription repression. The missense change identified in the current study is localized in the C2H2 type domain, which is a highly conserved motif in the ZNF589 protein involved in transcriptional regulation by interacting with different cellular molecules [25], therefore it is predicted that this variant would likely disrupt the function of the protein at the cellular level.

Notably, mutations in other C2H2-type zinc finger proteins have been reported before in relation to ID, including *ZNF526* (one patient), *ZNF41* (4 patients) [26] and *ZNF674* (1 family) [27], which supports the pathogenic role of the variant in *ZNF589* encountered in this study.

In the family MRQ15, the *de novo* variant which was found in the two affected members was c.1158G>C; p.(Trp386Cys); NM_001122834.2 in *HHAT* (hedgehog acyltransferase), which was located in MBOAT domain of the HHAT protein. The change has a high phyloP score of 5.433 as well as a high Grantham distance of 215. The substitution was not found in any of the unaffected members but it was present only in the two affected children as a heterozygous change. It is proposed that this variation among the affected members of MRQ15 has occurred as a result of a *de novo* germline change, which is not uncommon. Previously Rauch et al. [28] and de Light et al. [29], have reported a number of *de novo* variants in known and novel ID genes.

*HHAT*, belongs to the hedgehog family of gene, which is also referred to as skinny hedgehog. It is the precursor of an enzyme that acts within the secretory pathway to catalyze amino-terminal palmitoylation of hedgehog. It encodes a glycoprotein that undergoes autoproteolytic cleavage to generate its active form, the lipid modification is required for multimerization and distribution of hedgehog proteins. Defects in this protein could lead to improper signaling of shh and can lead to multiple defects including neural tube defects [30] as hedgehog proteins are involved in cell growth, survival and pattern of almost every plan of vertebrate body and has a major role in development of forebrain and midbrain [31]. Dennis et al. [32] have shown *HHAT* to be the candidate gene for congenital human holoprosencephaly, functional assays demonstrated that defects in *HHAT* could diminish secretion of hedgehog proteins. This defect in secretion can lead to abnormal patterning and extensive apoptosis within the craniofacial primordial leading to the structural defects in holoprosencephaly [32]. The role of *HHAT* in cognition is supported by the findings of Das et al. [33], where they treated trisomy 21 mice with Sonic hedgehog agonist and the treatment resulted in behavioral improvements and normalized performance in the Morris Water Maze task for learning and memory, the effect was due to improvement in cerebellar development and hippocampal function. Pan et al. [34] and Kyttala et al. [35] reported the role of shh signaling in primary cilia function. Ciliary defects have already been reported to be causative of many human syndromes involving ID as a clinical feature, such as bardet-biedl syndrome, holoprocencephaly, Kartagener syndromes, polycystic kidney disease, and retinal degeneration. These studies could possibly support the current findings in which only a heterozygous *de novo* change in *HHAT* was identified in the two affected siblings, the other allele being normal, hence the variation did not cause any structural malformation in both the siblings but the brain cognitive function was severely affected, which resulted in speech impairment in them.

### Conclusion

In conclusion, the identification of probable pathogenic variations in *KMT2B*, *ZNF589* and *HHAT* in the current study suggests potential importance of the particular pathways associated with these genes involved in cognitive dysfunctioning. In the absence of functional data the definitive role of variations in these novel genes cannot be ascertained without any doubt. However, after exome sequencing and segregation analysis of all the filtered variants, the currently reported were the only variants that segregated with the phenotype in the families. Therefore, it is proposed that these variations could be the most likely cause of ID in the studied families. The finding of additional variations in these genes in other ID families could validate the current results. In addition, functional studies could also define the role of these mutations. The current findings point to the possibility that there are many more unknown pathogenic genes in the known pathways for ID, which are yet to be identified.

## Supporting Information

Table S1 Selected homozygous and compound heterozygous variants for the family MRQ14 and polymerase chain reaction conditions.
(DOC)

Table S2 Family MRQ11 selected homozygous and compound heterozygous variants and polymerase chain reaction conditions.
(DOC)

Table S3 Selected homozygous and compound heterozygous variants of family MRQ15 and polymerase chain reaction conditions.
(DOC)

Table S4 Homozygous regions obtained among the affected members of family MRQ14 and MRQ11.
(DOC)
