# Supplementary material for: Exome Sequencing Identifies Three Novel Candidate Genes Implicated in Intellectual Disability
Source: PLoS One. 2014 Nov 18;9(11):e112687. doi: 10.1371/journal.pone.0112687 (PMC4236113; doi:10.1371/journal.pone.0112687)
Supplement: Table S1 — Selected homozygous and compound heterozygous variants for the family MRQ14 and polymerase chain reaction conditions. (DOC) [file pone.0112687.s001.doc]

Table S1. Selected homozygous and compound heterozygous variants for the family MRQ14, primer sequences, product sizes and annealing temperatures.

|  | **Gene** | **Exon** | **Sequence 5' - 3'** | **Product size** | **Annealing temperature** |
| --- | --- | --- | --- | --- | --- |
|  | *NME7* | 3 | F-TGTAAGGACTACTTTGCTGAAATG | 167bp | 58 ⁰C |
|  |  |  | R-TGTGCTAAGTGCCACAGGAG |  |  |
|  | *PPP1R9A* | 2 | F-TCGGAAATTGTTGGATTGC | 188bp | 58 ⁰C |
|  |  |  | R-TCTTGAATCCCTTACAAAAACTCC |  |  |
|  | *DYM* | 11 | F-AGGTTTTACCAATTCTTGAGATTC | 181bp | 58 ⁰C |
|  |  |  | R-GGGACATGTCTGTTCTGTTAACTC |  |  |
|  | *DLG1* | 8 | F-TCTGAGCTGCTTTTCTTTTCC | 212bp | 58 ⁰C |
|  |  |  | R-GCCAATTTTATGAACTGGGAAC |  |  |
|  | *MLL4 (KMT2B)* | 3 | F-TCTGGGGTAGAGGAGAAGATG | 197bp | 58 ⁰C |
|  |  |  | R-GCTGGAAAGTGTCCAAGGAG |  |  |
|  | *EHMT2* | 11 | F-CCTCTTTTCCTCCATCCAAG | 171bp | 58 ⁰C |
|  |  |  | R-ACCTCTCCGTCCACACTCTC |  |  |
|  | *SMEK1* | 15 | F-AGCCCAAGTTTCAAGCTTTC | 195bp | 58 ⁰C |
|  |  |  | R-GCAAAATGGGAGTCAAGAATG |  |  |
|  | *PLCD4* | 13 | F-GCTGTGGCTATGTGCTGAAG | 203bp | 58 ⁰C |
|  |  |  | R-GCTATCCGGCCTCTACATTC |  |  |
|  | *VAV2* | 29 | F-GGTACAGAGCAGGGTTCTGG | 394bp | 58 ⁰C |
|  |  |  | R-TGCAGAGGGTGTCTGGAAC |  |  |
|  | *ZNF227* | 6 | F-CAGAGAATTCACCCAGGAGAG | 207bp | 58 ⁰C |
|  |  |  | R-TTCCTCGCATTTATAGGGTTTC |  |  |
|  | *UGT8* | 2 | F-GGAGATTGACAGCAATCGAAC | 221bp | 58 ⁰C |
|  |  |  | R-ACCCACTTCAGCAGGATACC |  |  |
|  | *NAA15* | 17 | F-AAGGAGGCCTCTTATCCAATG | 210bp | 58 ⁰C |
|  |  |  | R-ATACCGTCACCATCAACCAC |  |  |
|  | *DNAH17* | 78 | F-GTGGGCTCTTTGCAGTGAC | 243bp | 58 ⁰C |
|  |  |  | R-TGACCCATGTTTACCTTCAGC |  |  |
|  | *DNAH17* | 76 | F-CTACCCGCTGTCATCCTTC | 255bp | 58 ⁰C |
|  |  |  | R-GAGGCACGAGCCTTCATTAC |  |  |
|  | *SACS* | 10 | F-TTGAATCATTTGATGTCCCAAG | 229bp | 58 ⁰C |
|  |  |  | R-CAGGTACACAACCAATCACCTC |  |  |
|  | *SACS* | 10 | F-GACCCAGCTGCTCTCTTTG | 213bp | 58 ⁰C |
|  |  |  | R-GTCCTGGATTTCTGACAGC |  |  |
|  | *TEP1* | 24 | F-CGCCTTCTTCAGGACACAG | 162bp | 58 ⁰C |
|  |  |  | R-TGTGCCTTACCTCCTTAGCC |  |  |
|  | *TEP1* | 12 | F-TCATACAGCATTGCCCTTTC | 170bp | 58 ⁰C |
|  |  |  | R-TCAGCTTCTCCCTCTTGAGC |  |  |

F, forward primer; R, reverse primer
