# Supplementary material for: Exome Sequencing Identifies Three Novel Candidate Genes Implicated in Intellectual Disability
Source: PLoS One. 2014 Nov 18;9(11):e112687. doi: 10.1371/journal.pone.0112687 (PMC4236113; doi:10.1371/journal.pone.0112687)
Supplement: Table S2 — Family MRQ11 selected homozygous and compound heterozygous variants and polymerase chain reaction conditions. (DOC) [file pone.0112687.s002.doc]

**Table S2. Family MRQ11 selected homozygous and compound heterozygous variants, primer sequences, product sizes and annealing temperatures.**

| **Gene** | **Exon** | **Sequence 5' - 3'** | **Product size** | **Annealing temperature** |
| --- | --- | --- | --- | --- |
| *SMOX* | 7 | F-GTGCTGTTTTCCGGTGAG | 177bp | 58 ⁰C |
|  |  | R-GGCTGGAGGTCACGAGTTAG |  |  |
| *TAS1R2* | 3 | F-TGCGCCAGAACTTCACTG | 261bp | 58 ⁰C |
|  |  | R-TGGTGTTGAAGGACAAGGTG |  |  |
| *ATP11A* | 2 | F-TGGGCAAGAGCTTTTGATG | 204bp | 58 ⁰C |
|  |  | R-CAGACCCAAGCCAAGTTACC |  |  |
| *ADORA2B* | 2 | F-CTGCTGCCTTGTGAAGTGTC | 291bp | 58 ⁰C |
|  |  | R-GCTGGCTGGAAAAGAGTGAC |  |  |
| *ZNF589* | 14 | F-TATGTCTGCGGAGAGTGTGG | 168bp | 58 ⁰C |
|  |  | R-CGATTTCTCCCTTGTGTGTG |  |  |
| *ZNF502* | 4 | F-TGGGAAAACATTTCGATGTC | 179bp | 58 ⁰C |
|  |  | R-ATGCCTTCCCACACCTATTG |  |  |
| *ADHFE1* | 10 | F-AGGAATTGCCAATGTTGATG | 174bp | 58 ⁰C |
|  |  | R-AAATGAAATCTCAGACTTGCTCAC |  |  |
| *ADHFE1* | 13 | F-GACTGAACTCCACCCAGAGC | 161bp | 58 ⁰C |
|  |  | R-GGCCATCATCAACATCCAG |  |  |
| *CMYA5* | 2 | F-GAAGGTGCTGGCAGAAAAAC | 206bp | 58 ⁰C |
|  |  | R-CACCTTTCTCCTTTTCAGAA |  |  |
| *CMYA5* | 2 | F-AGAAACACCGCCATATTTGC | 208bp | 58 ⁰C |
|  |  | R-CTCCAAGTGGTGCTTTGAAA |  |  |
| *DCHS1* | 6 | F-AGGCCATGTACGGCTTATG | 216bp | 58 ⁰C |
|  |  | R-ACCTGAGTTCCAGCAGTGG |  |  |
| *DCHS1* | 2 | F-CATCTTTCTCAGTGCAGATGG | 245bp | 58 ⁰C |
|  |  | R-CTGTGGCTGTAACCCTCAAG |  |  |
| *DPAGT1* | 7 | F-TTCAAATAGTGGCCCAGTCA | 219bp | 58 ⁰C |
|  |  | R-CCCCTTGGATCTGATGTAGG |  |  |
| *DPAGT1* | 1 | F-CTTGCCCGTTACCTGAAGAG | 190bp | 58 ⁰C |
|  |  | R-TTGAGGTCCTGACCACAGAG |  |  |
| *DENND2C* | 7 | F-ACAGAGCAAGACCCCATCTC | 238bp | 58 ⁰C |
|  |  | R-TTCGTGTTTGCAACTTCACC |  |  |

F, forward primers; R, reverse primers
