# Supplementary material for: Exome Sequencing Identifies Three Novel Candidate Genes Implicated in Intellectual Disability
Source: PLoS One. 2014 Nov 18;9(11):e112687. doi: 10.1371/journal.pone.0112687 (PMC4236113; doi:10.1371/journal.pone.0112687)
Supplement: Table S3 — Selected homozygous and compound heterozygous variants of family MRQ15 and polymerase chain reaction conditions. (DOC) [file pone.0112687.s003.doc]

Table S3. Selected homozygous and compound heterozygous variants of family MRQ15, primer sequences, product sizes and annealing temperatures.

| **Gene** | **Exon** | **Sequence5' - 3'** | **Product size** | **Annealing Temperature** |
| --- | --- | --- | --- | --- |
| *SF3B3* | 3 | F-CTTAGCCTCCCGAGTAGCTG | 273bp | 58 ⁰C |
|  |  | R-GCCTAAAGGCCATGAGTGAC |  |  |
| *LARGE* | 3 | F-AAGCCCGTGTCTCTGTCAC | 231bp | 58 ⁰C |
|  |  | R-AAGGTTCTCGCTGTCTCCAG |  |  |
| *HHAT* | 9 | F-GCAGCCCTCTGTATGTCTCC | 259bp | 58 ⁰C |
|  |  | R-AAGGATCCTGCTCACCAGAC |  |  |
| *NEDD4* | 1 | F-CCGGAGATGACTTGGATAGC | 330bp | 58 ⁰C |
|  |  | R-TCAATTCATTATCGACCACAGC |  |  |
| *PLCH1* | IVS13+1C>T | F-AAAGCCCAGCTCATTCTCTG | 157bp | 58 ⁰C |
|  |  | R-CCCTCCTTGCCAGTACTCTG |  |  |
| *PDS5B* | IVS4+1C>T | F-AGTCATTGTGGTGAACTGTTGG | 214bp | 58 ⁰C |
|  |  | R-ATGTGCCAAAAGGTGAATTG |  |  |
| *WASF1* | IVS25-1G>A | F-GTGCAATATTCAGTTTTATTTTGG | 258bp | 58 ⁰C |
|  |  | R-TGCTTCCTCTTTTCCTTCCTC |  |  |
| *DENND2A* | 1 | F-GAGTCAGGCTTCAGAAGGAGTC | 231bp | 58 ⁰C |
|  |  | R-TTTCTTCCCTTCCCATTCTG |  |  |
| *HMCN1* | 46 | F-AGGCAACTAATATTTCCATTCC | 182bp | 58 ⁰C |
|  |  | R-AGGCCACCCATCTTTGAAC |  |  |
| *HMCN1* | 85 | F-TGCAATCCTGAATTGTGAGG | 192bp | 58 ⁰C |
|  |  | R-ACAGAGGGGTATTGGCTGTG |  |  |
| *MED13L* | 10 | F-GGCTTAAATGGGACGCTAAC | 164bp | 58 ⁰C |
|  |  | R-ACTGCCACAGGGAAATCATC |  |  |
| *MED13L* | 6 | F-TGTAGTGAGCATTTGTCCTGTG | 150bp | 58 ⁰C |
|  |  | R-TTGAAATGGTGCAGGTGAAG |  |  |
| *ZNF772* | 5 | F-CATATGTGGGAAAGTTTTTAAT | 227bp | 58 ⁰C |
|  |  | R-GAACACTCCAATGTTTAATGAG |  |  |
| *ZNF772* | 5 | F-GCAACACCAAGTGTGAGGAG | 235bp | 58 ⁰C |
|  |  | R-TTCCCACATATGCCACACTC |  |  |

F, forward primer; R, reverse primer
