# Supplementary material for: Exome Sequencing Identifies Three Novel Candidate Genes Implicated in Intellectual Disability
Source: PLoS One. 2014 Nov 18;9(11):e112687. doi: 10.1371/journal.pone.0112687 (PMC4236113; doi:10.1371/journal.pone.0112687)
Supplement: Table S4 — Homozygous regions obtained among the affected members of family MRQ14 and MRQ11. (DOC) [file pone.0112687.s004.doc]

Table S4: Homozygous regions obtained among the affected members of family MRQ14 and MRQ11.

| **Family MRQ14** | | | |  |
| --- | --- | --- | --- | --- |
| **Chromosome** | **Flanking SNP** | **Boundaries (MBs)** | **Size (MBs)** | **Ranking** |
| Chromosome 19 | rs16966190; rs41334244 | 32,19-45,45 | 13.2 | 1 |
| Chromosome 18 | rs17573901; rs2155957 | 36,34-48,69 | 12.3 | 2 |
| Chromosome 1 | rs643927; rs4617427 | 91,66-99,18 | 7.5 | 3 |
| Chromosome 4 | rs1397933; rs11733857 | 40,18-40,93 | 0.7 | 4 |
| **Family MRQ11** | | | |  |
| Chromosome 13 | rs973907; rs7322187 | 91,72-105,1 | 13.3 | 1 |
| Chromosome 3 | rs3774389; rs2236975 | 42,26-50,48 | 8.2 | 2 |
| Chromosome 2 | rs10185886; rs6755276 | 26,07-33,94 | 7.8 | 3 |
| Chromosome 3 | rs3774589; rs7636357 | 53,81-60,71 | 6.3 | 4 |

SNP, single nucleotide polymorphism; MBs, mega bases, All the positions are obtained from Human build hg19
